# Characterization of Dnmt1 Binding and DNA Methylation on Nucleosomes and Nucleosomal Arrays

**DOI:** 10.1371/journal.pone.0140076

**Published:** 2015-10-23

**Authors:** Anna Schrader, Thomas Gross, Verena Thalhammer, Gernot Längst

**Affiliations:** Institute of Biochemistry III, University of Regensburg, Regensburg, Germany; Inc, UNITED STATES

## Abstract

The packaging of DNA into nucleosomes and the organisation into higher order structures of chromatin limits the access of sequence specific DNA binding factors to DNA. In cells, DNA methylation is preferentially occuring in the linker region of nucleosomes, suggesting a structural impact of chromatin on DNA methylation. These observations raise the question whether DNA methyltransferases are capable to recognize the nucleosomal substrates and to modify the packaged DNA. Here, we performed a detailed analysis of nucleosome binding and nucleosomal DNA methylation by the *maintenance* DNA methyltransferase Dnmt1. Our binding studies show that Dnmt1 has a DNA length sensing activity, binding cooperatively to DNA, and requiring a minimal DNA length of 20 bp. Dnmt1 needs linker DNA to bind to nucleosomes and most efficiently recognizes nucleosomes with symmetric DNA linkers. Footprinting experiments reveal that Dnmt1 binds to both DNA linkers exiting the nucleosome core. The binding pattern correlates with the efficient methylation of DNA linkers. However, the enzyme lacks the ability to methylate nucleosomal CpG sites on mononucleosomes and nucleosomal arrays, unless chromatin remodeling enzymes create a dynamic chromatin state. In addition, our results show that Dnmt1 functionally interacts with specific chromatin remodeling enzymes to enable complete methylation of hemi-methylated DNA in chromatin.

## Introduction

In mammals, DNA methylation occurs at CpG sites of which 60–80% are modified in a cell-type specific pattern and is generally associated with repressed states of chromatin [1–5]. DNA methylation is involved in epigenetic processes such as differentiation, proliferation, transcriptional regulation, genomic imprinting, X-chromosome inactivation, silencing of repetitive elements, maintenance of genomic stability and DNA repair [3,4]. Although some functional overlap exists [6], the DNA methyltransferases can be generally divided into two classes: The *maintenance* DNA methyltransferase Dnmt1 maintains methylation patterns on the newly synthesized daughter strands during replication, and the *de novo* DNA methyltransferases Dnmt3a and Dnmt3b introduce novel methylation marks in the genome [7].

The fundamental unit of chromatin is the nucleosome, composed of 147 bp of DNA wrapped around an octamer of H2A, H2B, H3 and H4 core histone proteins. Nucleosomes impose a significant barrier for sequence-specific recognition, impeding the access of regulatory proteins to DNA. Chromatin presents the natural substrate for DNA dependent processes like control of gene expression, DNA replication, recombination and repair [8]. The enzymatic properties of the DNA methyltransferases have been extensively studied on free DNA as substrate [9–14]. However, functional studies on DNA methylation in chromatin are still limited. Nucleosomal DNA methylation was either shown not to be affected by Dnmt1 irrespective of the DNA sequence [15], or to be significantly reduced for Dnmt1 [16,17]. It was recently shown that in cells DNA methylation occurs mainly in the accessible linker regions, suggesting that nucleosomes stably occupy their underlying DNA sequence and that Dnmt1 is only capable to target the linker regions *in vivo* [18,19]. Cells evolved ATP-dependent chromatin remodeling complexes that hydrolyze ATP to alter the underlying chromatin structure and to regulate DNA accessibility [8]. Deletion of the remodeling ATPase *DDM1* in *Arabidopsis* or its murine homolog *LSH* results in a global loss of DNA methylation [20,21], showing the impact of remodeling enzymes on DNA methylation. Lsh was shown to recruit *de novo* DNA methyltransferases [22] and to cooperate with Dnmt3b in polycomb repressive complex—and histone deacetylase—mediated gene silencing [23,24]. Mutations in the *ATRX* gene, belonging to the Rad54 subfamily of Snf2 ATPases, [25] result in the ATR-X syndrome that is characterized by both hypermethylation of repeated heterochromatin regions and hypomethylation of ribosomal DNA (rDNA) repeats [26]. The *de novo* DNA methyltransferases Dnmt3a and Dnmt3b were found in complexes with Brg1 [27], and the human Snf2h containing complex NoRC [28] represses rDNA transcription by recruiting Dnmt1 and histone deacetylases [29]. A direct interaction of Dnmt1 with human Snf2h increases the affinity of Dnmt1 towards nucleosomes [16], and recent genome wide studies suggest that nucleosomal DNA methylation may be enriched during cellular differentiation [30].

In order to study the potential of Dnmt1 to methylate DNA reconstituted in chromatin, we characterized its DNA and nucleosome binding ability and the DNA methylation efficiency on reconstituted nucleosomes and nucleosomal arrays. The methyltransferase activity of Dnmt1 is strongly inhibited by nucleosomes, and its binding to chromatin requires the presence of linker DNA. Even though Dnmt1 has an intrinsic DNA length sensing property, binding with highest affinity to the longest DNA molecules and significantly binding only to DNA of 20 bp and longer, it is able to bind nucleosomes with short DNA linkers. However, the linkers have to be present symmetrically on both sides of the nucleosome core for efficient binding. Correspondingly we show the simultaneous binding of Dnmt1 to both DNA linkers exiting the nucleosome core. The binding pattern correlates well with the efficient methylation of the DNA linkers and a sharp drop of methylation efficiency at the DNA-nucleosome core boundary. Using nucleosomal arrays, reconstituted on non-methylated and hemi-methylated DNA, we show that chromatin remodeling is a prerequisite of efficient DNA methylation in chromatin. Interestingly, we observed a functional interaction between Dnmt1 and the chromatin remodeling complex ACF on hemi-methylated DNA, suggesting an active mechanism that efficiently converts hemi-methylated DNA into symmetrically methylated sites.

## Results

### Dnmt1 requires symmetric DNA linkers to bind to nucleosomal DNA

Full-length human Dnmt1 was expressed in insect cells after baculovirus infection and purified via its 6x-His-tag (S1A Fig). The recombinant enzyme exhibits the expected high DNA methylation activity on hemi-methylated DNA substrates, but still shows significant methylation activity on non-methylated DNA (S1B Fig). The activity data fits well with the previously published activities that range from 2 to 200 fold preferences of Dnmt1 for hemi-methylated DNA methylation [13,31].

**Fig 1:**
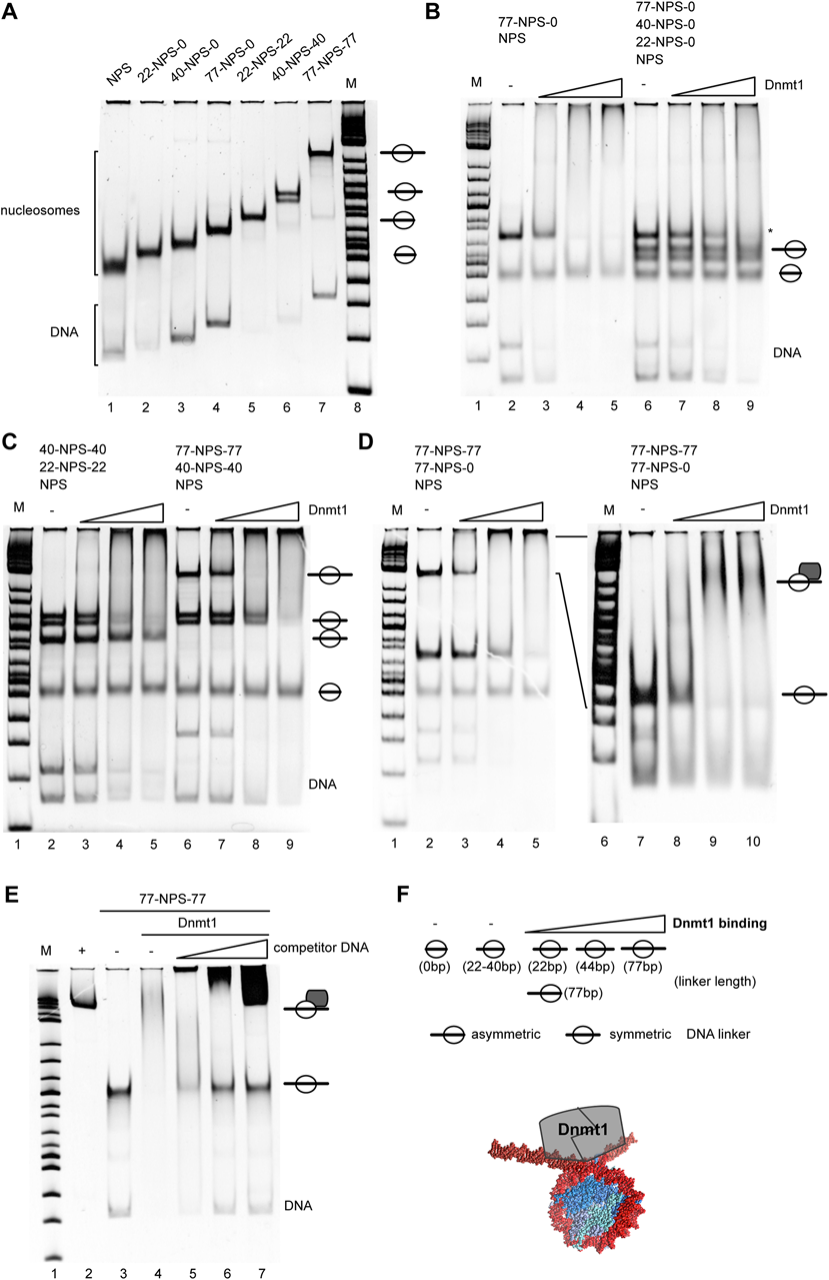
Dnmt1 requires symmetric linker DNA in order to bind to nucleosomal DNA. **(A)** DNA fragments containing the nucleosome positioning sequence (NPS) located either in the center or at the border of the DNA molecules were assembled into mono-nucleosomes using the salt dialysis method. The DNA templates are named according to the size and location of the DNA linkers with respect to the NPS. **(B, C)** Different combinations of nucleosomal substrates (50 nM each) were mixed in a 1:1 ratio (lanes 2 and 6) and incubated with increasing concentrations of Dnmt1 (100 nM– 500 nM; lanes 3–5 and 7–9). Reactions were analyzed by native polyacrylamide gel electrophoresis next to a molecular weight marker (M). **(D)** Monitoring the Dnmt1 binding affinity to mono-nucleosomes containing asymmetrical and symmetrical DNA linkers (77-NPS-0 and 77-NPS-77). Reactions were analyzed on 6% (left panel) and on 4.5% (right panel) native polyacrylamide gels. The position of the Dnmt1-nucleosome complex is indicated (lanes 7–10). **(E)** Binding of Dnmt1 does not disrupt the nucleosome. The 77-NPS-77 nucleosome (lane 3) was incubated with Dnmt1 to form the stable nucleosome–Dnmt1 complex (lane 4). This complex was incubated with increasing concentrations of competitor DNA to compete Dnmt1 off the nucleosome (lane 5–7). Reaction products were analyzed on a native polyacrylamide gel. The positions of nucleosomes and Dnmt1-nucleosome complexes are indicated. Lane 2 shows the 6 kb competitor plasmid DNA. **(F)** (upper part) Summary of the results of the Dnmt1 band shift assays and (lower part) a cartoon showing how Dnmt1 (grey box) could bind on the entry/exit sites of the nucleosome (red: DNA; bluish: histones).

To characterize the constraints of Dnmt1 dependent nucleosome binding, defined nucleosomal substrates harbouring a single positioned nucleosome were used in electro mobility shift assays (EMSAs). The DNA fragments of varying length contained the 601 nucleosome positioning sequence (referred to as NPS) [32]. The NPS was extended at only one side (asymmetric nucleosomes containing only one linker) or at both sides (symmetric nucleosomes with two DNA linkers) with DNA linkers of 0 to 77bp in length (Fig 1A and S2 Fig). DNA fragments (S1 Table) were generated by polymerase chain reactions (PCR) and reconstituted into nucleosomes by the salt dialysis method [18]. The binding studies with increasing concentrations of Dnmt1 were performed using a mixture of different DNA and nucleosome substrates, and do directly visualize the preferential binding substrates of Dnmt1 (Fig 1B–1D).

Performing the electromobility shift assays with nucleosomes either lacking DNA linkers or possessing asymmetric linkers of 22, 40 and 77 bp showed that Dnmt1 only binds significantly to nucleosomes harbouring the asymmetric long linker (77 bp) and not to the other substrates (Fig 1B). This result demonstrates that Dnmt1 does not recognize the nucleosome core, and significant binding is only observed with a linker length of more than 40 bp. As revealed by the EMSA, the nucleosome with a linker of 77 bp is bound with similar efficiency as the free DNA (Fig 1B), suggesting that Dnmt1 preferentially recognizes the free DNA. This result implies that Dnmt1 is not capable to recognize chromatin *in vivo*, as the linker lengths vary between 30 and 50 bp in mammalian cells [33]. Thus we tested whether the natural substrate of Dnmt1 is a nucleosome with DNA linkers exiting at both sides of the nucleosome core. Surprisingly, if nucleosomes with symmetrical DNA linkers were used, a binding of Dnmt1 to substrates with 40 and 22 bp long linkers was observed (Fig 1C). This suggests a bipartite binding mode of Dnmt1, being able to simultaneously recognize the linker DNA at both sides of the nucleosomes. Accordingly, Dnmt1 would cross the nucleosomal dyad to access both DNA linkers (Fig 1F). To address whether Dnmt1 forms specific Dnmt1-nucleosome complexes, electromobility shift assays were performed using low percentage polyacrylamide gels (Fig 1D). Staining the gel with ethidium bromide reveals a discrete nucleoprotein complex. The binding of Dnmt1 to the nucleosome does not disrupt the octamer as the addition of competitor DNA after complex formation results in the re-appearance of the intact nucleosomal bandshift (Fig 1E). In summary, the results of the interaction assays indicate specific binding of Dnmt1 to linker DNA exiting at both sides of the nucleosome core, without disrupting the octameric structure.

### DNA length and DNA–modification dependent binding of Dnmt1

**Fig 2:**
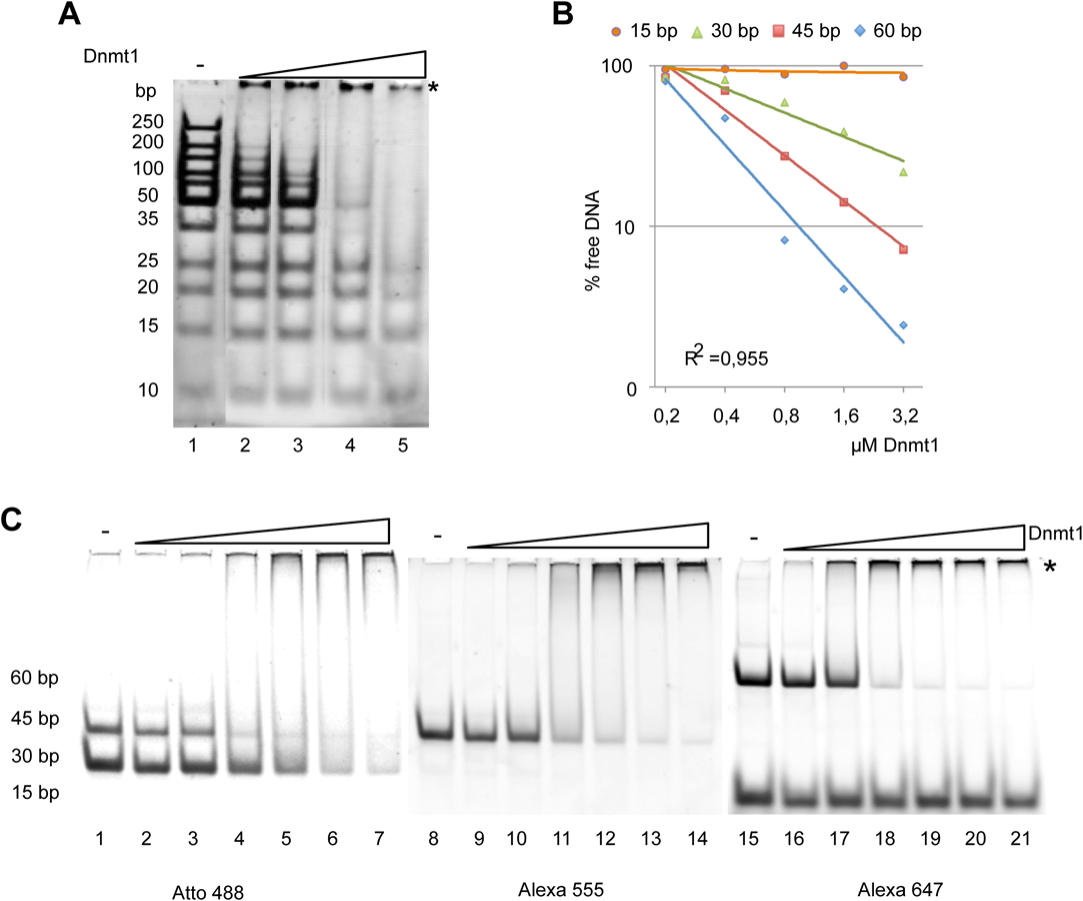
DNA binding properties of Dnmt1. **(A)** DNA fragments of different length (size range from 15 to 250 bp) were incubated with increasing concentrations of Dnmt1 (lanes 2–5). DNA and nucleoprotein complexes were separated on native polyacrylamide gels and stained with ethidium bromide. The asterisk indicates the positions of the Dnmt1-DNA complexes. **(B)** Quantification of the Dnmt1 binding assay shown in **(C)**, using an equimolar mixture of fluorescently labeled DNA fragments from 15 to 60 bp in length. The remaining free DNA was quantified and plotted. **(C)** Competitive electromobility shift assay with a mixture of differently sized fluorescently labeled double stranded (ds) oligonucleotides (4 pmol each). The DNA fragments (15–60 bp; 30 bp: lane 2–7, 45 bp: lane 8–14; 15 bp and 60 bp: lane 15–21) were incubated with increasing concentrations of Dnmt1 (0.1 μM- 0.5 μM, and complex formation was analyzed on a 15% native polyacrylamide gel.

As already mentioned our studies indicate that Dnmt1 preferentially binds to the free DNA. Therefore we characterized its DNA binding properties in detail. In order to estimate the minimal DNA length required for Dnmt1 binding, increasing concentrations of the protein were incubated with a mixture of DNA fragments of different length (100 bp DNA ladder, Thermo Scientific). Surprisingly, this competitive binding experiment reveals a DNA length sensing activity of Dnmt1, as the enzyme preferentially binds to long DNA fragments, and only with increasing protein concentrations does progressively bind to shorter DNA fragments (Fig 2A). The minimal DNA size bound by Dnmt1 in this assay was 20 bp. These results were confirmed by equimolar mixing of the fluorescently labeled DNA fragments of 15, 30, 45 and 60 bp, showing the preferential binding of the longer DNA fragments and no binding of the 15 bp long DNA fragment (Fig 2B and 2C). Our data suggest that the DNA binding domain of Dnmt1 requires at least 20 bp of DNA to display a significant binding affinity. The DNA length sensing property suggests a cooperative binding mode and/or hints to the existence of multimeric forms of Dnmt1 in solution.

**Fig 3:**
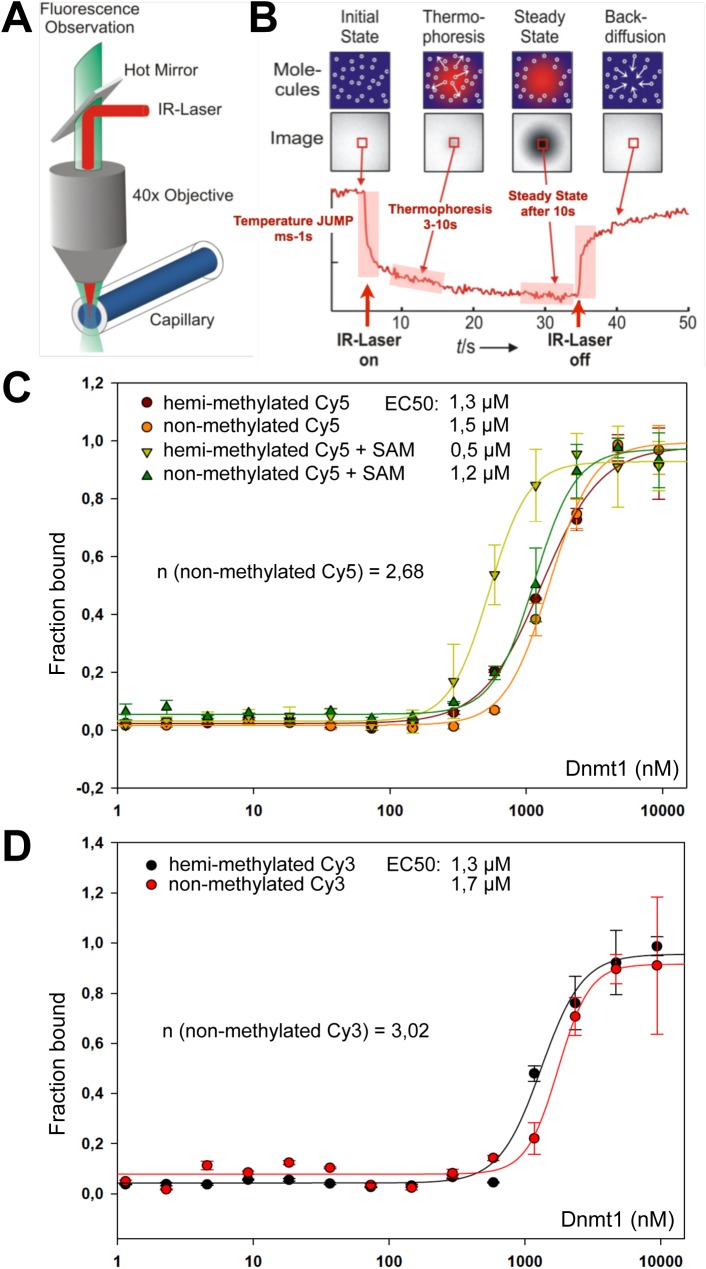
Dnmt1 exhibits similar binding affinities towards hemi- and non-methylated DNA. **(A)** Scheme showing the setup of a microscale thermophoresis (MST) assay. The aqueous solution inside the capillary is locally heated with a focused IR-laser and coupled with an epifluorescence microscope by using an IR mirror. **(B)** MST is based on the directed movement of molecules along temperature gradients, an effect termed thermophoresis. The fluorescence inside the capillary is measured for each different concentration of the unlabeled molecule, and the normalized fluorescence in the heated spot is plotted against time. The IR laser is switched on at t = 5 s, the fluorescence decreases as the temperature increases, and the labeled molecules move away from the heated spot because of thermophoresis. When the IR laser is switched off, the molecules diffuse back. The thermophoretic movement for a single ligand concentration is shown in **(B)**. **(C, D)** Quantitative analysis of Dnmt1 binding to hemi- and non-methylated DNA by MST. The indicated fluorescently labeled DNA substrate of 60 bp in length was incubated with increasing concentrations of Dnmt1 (300 nM—9.4 μM), in the presence or absence of SAM. Binding curves were normalized to the fraction of bound molecules. All MST curves were fitted using the Hill-equation, and EC50 values as well as Hill coefficients (n) are given. **(C)** shows the analyses of the measurements for the Cy5 labeled DNA substrates, **(D)** for the Cy3 labeled DNA substrates.

Since we were interested in quantifying the binding affinities and to reveal a potential cooperative mode of binding, we performed microscale thermophoresis experiments (MST), a technique that is based on the movement of molecules in temperature gradients [34,35] (Fig 3A and 3B). The thermophoretic mobility of fluorescently labeled DNA was recorded at different Dnmt1 concentrations ranging from 0 to ~10 μM, and the data were used to calculate binding affinities. Dnmt1 exhibits a binding affinity of 1,5 μM towards the 60 bp long non-methylated DNA, and the reaction is highly cooperative, with a Hill coefficient (n) of about 2.7 (Fig 3C and 3D). The Hill coefficient is used to provide a quantitative measure of cooperativity of ligand binding with values of n > 1 indicating positive cooperativity. Positive cooperativity occurs when an enzyme has several binding sites, and the binding of one substrate molecule facilitates the binding of a second or more substrate molecules.

Surprisingly, Dnmt1 exhibits similar binding affinities to non- and hemi-methylated DNA, even though the protein methylates much more efficiently the hemi-methylated DNA (from 2 to 200-fold with respect to the model system) [31]. Our binding study suggests that catalytic properties and not the binding properties determine the efficient methylation of hemi-methylated DNA over non-methylated DNA. Still, we observe a twofold increase in the binding affinity of Dnmt1 towards hemi-methylated DNA in the presence of saturating amounts of the cofactor S-adenosylmethionine (SAM) (Fig 3C), suggesting specific structural changes in the enzyme to accommodate the hemi-methylated DNA in the active center of the enzyme. The differences in binding affinity in the presence of SAM may contribute to the observed difference in methylation efficiency. The MST data does also confirm the results of the competitive electromobility shift assays. The Hill coefficient of about 2.7 suggests cooperative binding of the substrate or the presence of multimeric forms of Dnmt1 in solution that could be responsible for the DNA length dependent binding activity.

With regard to potential multimerization of Dnmt1, we analyzed the apparent molecular weight of Dnmt1 by gelfiltration chromatography using different running conditions (S3A and S3B Fig). Indeed, Dnmt1 migrates with an apparent mass of 400 kDa that would correspond to a functional dimer. However, migration properties do not necessarily depend on the molecular mass. Therefore we denatured recombinant Dnmt1 by sodium dodecyl sulfate (SDS) and heat incubation (S3B Fig). Indeed, after denaturation we can observe Dnmt1 forms migrating with the apparent mass of a monomer, indicating that Dnmt1 exists as a stable dimer in solution. To further prove Dnmt1 dimerization, we expressed the targeting sequence (TS) domain of Dnmt1 fused to the maltose binding protein (MBP), and used it in co-immunoprecipitation experiments (S3C Fig). The incubation of Dnmt1 with the TS domain results in the specific interaction of the two proteins revealing that Dnmt1 has the capability to self-interact. Taken together, the competitive EMSAs, MST and dimerization assays suggest that Dnmt1 is binding cooperatively to DNA due to homotypic Dnmt1 interactions. The stability of the complex does also suggest that dimeric Dnmt1 associates with the nucleosome.

### Dnmt1 binds to the entry/exit sites of the nucleosome

**Fig 4:**
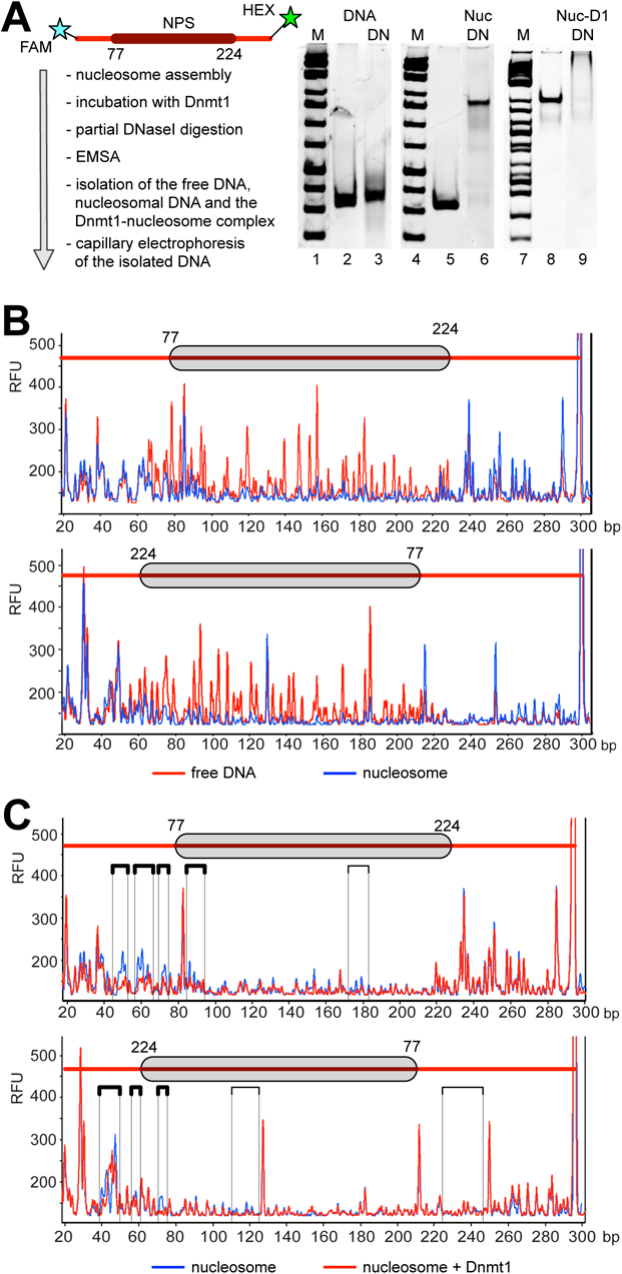
Dnmt1 binds to the DNA linkers at the entry/exit sites of the nucleosome. **(A)** Experimental setup (left) and quality analysis (right) of the DNaseI protection assay. The 77-NPS-77 template (301 bp long) was fluorescently labeled by PCR using 5’ labeled oligonucleotides (5'FAM and 5'HEX) and was assembled into nucleosomes by salt gradient dialysis. Free DNA (lanes 2, 3 and 5) and nucleosomes (Nuc; lanes 6 and 8) and nucleosome-Dnmt1 complexes (Nuc-D1; lane 9) were partially digested with 0.1U DNaseI (DN; lanes 3, 6 and 9). The reaction was stopped by the addition of 5 mM EDTA to inactivate the DNaseI and subsequently separated on a native polyacrylamide gel. DNaseI treated complexes were extracted from the gel and analyzed by capillary electrophoresis. **(B)** Comparison of the electropherograms of DNaseI treated free DNA (red line) with nucleosomal DNA (blue line). The position of the nucleosome core is indicated (grey bar). The electropherograms for both directions (5’HEX: on top and 5’FAM: on bottom) are shown. **(C)** As in **(B)**, the electropherograms of the DNase I treated nucleosome (blue line) are compared with the Dnmt1-nucleosome complex (red line). The electropherograms for both directions (5’HEX: on top and 5’FAM: on bottom) are shown. The protected regions are highlighted in boxes. RFU: relative fluorescence units.

To further elucidate the mode of Dnmt1-nucleosome interaction, we performed DNase I footprinting experiments. Free DNA, nucleosomes and nucleosome-Dnmt1 complexes were mildly treated with DNase I. The DNase I reaction was stopped with EDTA and the complexes were resolved on native polyacrylamide gels. DNase I was titrated such that the DNA started to exhibit a minor fraction of degradation products (Fig 4A). These DNase I conditions introduce about 1 nick per DNA template strand, allowing proper detection of the binding sites [36]. The free DNA or nucleosomes were incubated with or without Dnmt1, partially digested with DNase I and then subsequently loaded on a native polyacrylamide gel for separation. The individual bands corresponding to the DNA, nucleosome and nucleosome-Dnmt1 complex were isolated from the gel. The DNA was purified and analyzed on a capillary sequencer (Fig 4). Partial DNase I digestion of the nucleosomal DNA resulted in a characteristic cleavage pattern that differed from free DNA, demonstrating the efficient and localized assembly of the nucleosomes on the positioning sequence (Fig 4B). The NPS is located from position 77 to 224 on the DNA, and DNase I protections are detected in this area, extending about 10 to 15 bp left and right from the positioning sequence. Extended protection comes from interactions of the linker DNA with the histone tails and still weak interactions with the nucleosomal core. In addition, these extensions are explained by additional rotational positions around the center of the NPS sequence.

The analysis of the Dnmt1-nucleosome complex revealed additional DNase I protections in the linker DNA and at the DNA entry/exit sites, next to the positioned nucleosome (Fig 4C, boxes). Furthermore, weak changes in the DNase I footprinting profile were observed in the region of the dyad axis (weak changes, because the nucleosome already restricts DNase I accessibility). As the nucleosomal DNA was fluorescently labeled with 6-hexachlorofluorescein (HEX) and 6-carboxyfluorescein (FAM) at both ends of the DNA fragment (Fig 4C, upper and lower panel), we were able to monitor and internally control the binding of Dnmt1 by analyzing the footprinting pattern from both sides. The analysis revealed that Dnmt1 binds simultaneously to both entry/exit sites of the nucleosomes. Signals are most prominent in the size range of 20 to 150 bp, where the capillary electrophoresis has its best resolution. With larger fragment size the signals are compressed and loose their resolution.

The protection pattern at both entry/exit sites and the protection at the dyad axis suggest that Dnmt1 binds to both linker DNAs at the same time, thereby crossing the nucleosomal dyad axis. The data is supported by the electro mobility shift assays, also suggesting the simultaneous binding of Dnmt1 to both entry/exit sites of the nucleosome.

### Dnmt1 dependent methylation of nucleosomal DNA is strongly inhibited

**Fig 5:**
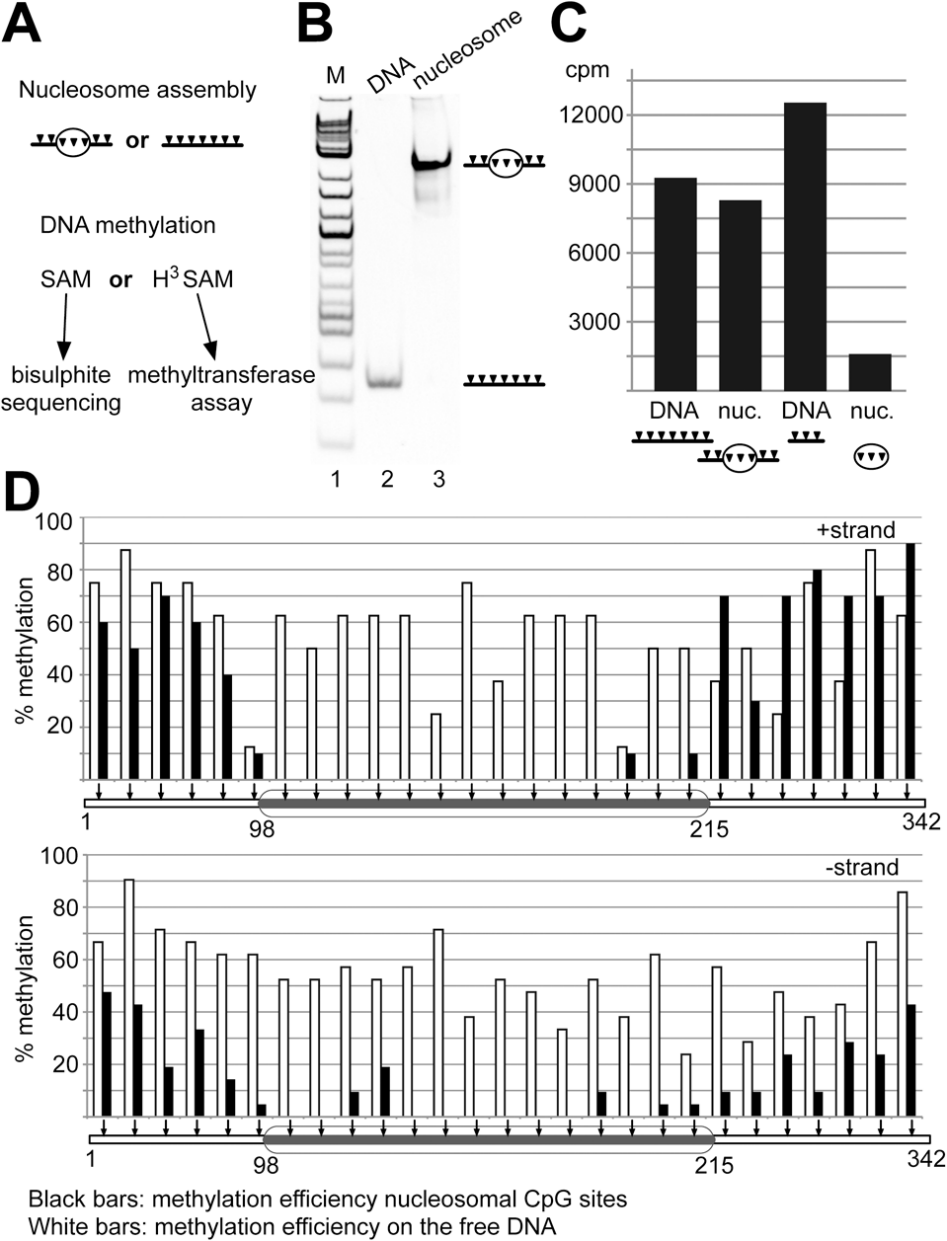
Nucleosomes inhibit the Dnmt1 dependent DNA methylation *in vitro*. **(A)** Experimental setup of the bisulfite sequencing and the radioactive methyltransferase assay. Black triangles indicate CpG sites and the oval indicates the position of the nucleosome. **(B)** Analysis of the C91-NPS2-C104 template (342 bp fragment harbouring 27 CpG sites) as free (lane 2) and nucleosomal DNA (lane 3) on a native polyacrylamide gel. **(C)** Enzymatic activity of Dnmt1 on the NPS2 (no linker DNA in nucleosomal form) and the C91-NPS2-C104 template both as free and nucleosomal DNA analyzed in the radioactive methyltransferase assay using [^3^H]-SAM (360 nM) as substrate. The incorporation of the [^3^H]-modified CH_3_ group was quantified, indicated as counts per minutes (cpm). **(D)** Bisulfite analysis of the DNA methylation reaction of Dnmt1, using the C91-NPS2-C104 DNA as free DNA and nucleosomal substrate. The DNA methylation efficiency of Dnmt1 at individual CpG sites of the free (white bars) and mononucleosomal DNA (black bars) is given. The (+) and (-) strands are shown and CpG sites are marked as arrows. The ellipse illustrates the position of the nucleosome. In both assays, the bisulfite sequencing and the radioactive methyltransferase assay, the C91-NPS2-C104 and NPS2 templates were use as as non-methylated substrates.

To evaluate the extent of Dnmt1 dependent DNA methylation activity on nucleosomes, two different templates with a modified 601 sequence (NPS2, C91-NPS2-C104) containing additional CpG sites were reconstituted into chromatin. CpG sites were placed such that potential methylation sites were spread along the whole nucleosome positioning sequence. Nucleosomes were reconstituted either on DNA fragments containing DNA linkers with CpG sites (C91-NPS2-C104), or lacking the linkers (NPS2). In contrast to the electromobility shift assays, histone:DNA ratios were titrated such that DNA was fully reconstituted into mononucleosomes (Fig 5B). Dnmt1 is only 4 times more active on hemi-methylated DNA (S1B Fig), when using 100 nM of protein concentration. Prolonged incubation times and high concentrations of Dnmt1 used in the activity assays shown in Fig 5 ensure the efficient methylation of the non-methylated nucleosomal and free DNA templates. DNA methylation reactions using ^3^H-SAM were performed with each template either as free DNA or reconstituted into mononucleosomes (Fig 5C). The incorporation of ^3^H-labeled methyl-groups was quantified by scintillation counting after the reaction. Performing the DNA methylation reaction with the free DNA and nucleosomes reconstituted on the 342 bp long C91-NPS2-C104 template revealed no major difference in DNA methylation efficiency (Fig 5C, lanes 1 and 2). The excess of free DNA linkers ensures efficient DNA methylation on this template. In contrast, when comparing the free DNA and the nucleosomal template reconstituted on the 150 bp DNA (NPS2), lacking accessible linker DNA, a strong inhibition of nucleosomal DNA methylation can be observed (Fig 5C, lanes 3 and 4). The data shows that the nucleosomal core particle does not serve as a substrate for Dnmt1. However, this assay does not tell whether there is remaining, low level DNA methylation in the nucleosome core. Moreover we showed in Fig 1 that Dnmt1 does not bind to nucleosomes without DNA linkers. Hence it is possible that lack of DNA methylation is a result of non-apparent binding of Dnmt1 towards the nucleosome core.

As we wanted to test whether the nucleosome core can be methylated, we performed non-radioactive DNA methylation assays using the 342 bp long C91-NPS2-C104 DNA fragment as free DNA and a nucleosomal substrate that is bound by Dnmt1. The DNA methylation assays with non-radioactive SAM were followed by bisulfite conversion and DNA sequencing. The treatment of DNA with bisulfite results in the deamination of the non-methylated cytosine residues to uracil. DNA templates were then amplified by PCR, cloned and the DNA methylation pattern was subsequently analyzed by sequencing of 50 individual clones. This technique allows a quantitative and qualitative evaluation of DNA methylation events on the (+) and (-) strand of DNA at the same time (Fig 5D). Visual inspection clearly shows that, compared to free DNA (white bars), the accessible linker DNA is efficiently methylated and that the nucleosomal DNA is highly refractory to methylation by Dnmt1. If nucleosomal DNA methylation events are observed, they locate to CpG sites close to the entry/exit sites of the nucleosomes and are not distributed along the entire nucleosome (Fig 5D).

The bisulfite analysis perfectly matches the radioactive DNA methylation assay in that the Dnmt1 dependent DNA methylation activity on positioned nucleosomes is strongly reduced, indicating that active mechanisms, like chromatin remodeling, are required to allow Dnmt1 to access DNA in chromatin.

### Specific targeting of DNA methylation to hemi-methylated chromatin by chromatin remodelers

**Fig 6:**
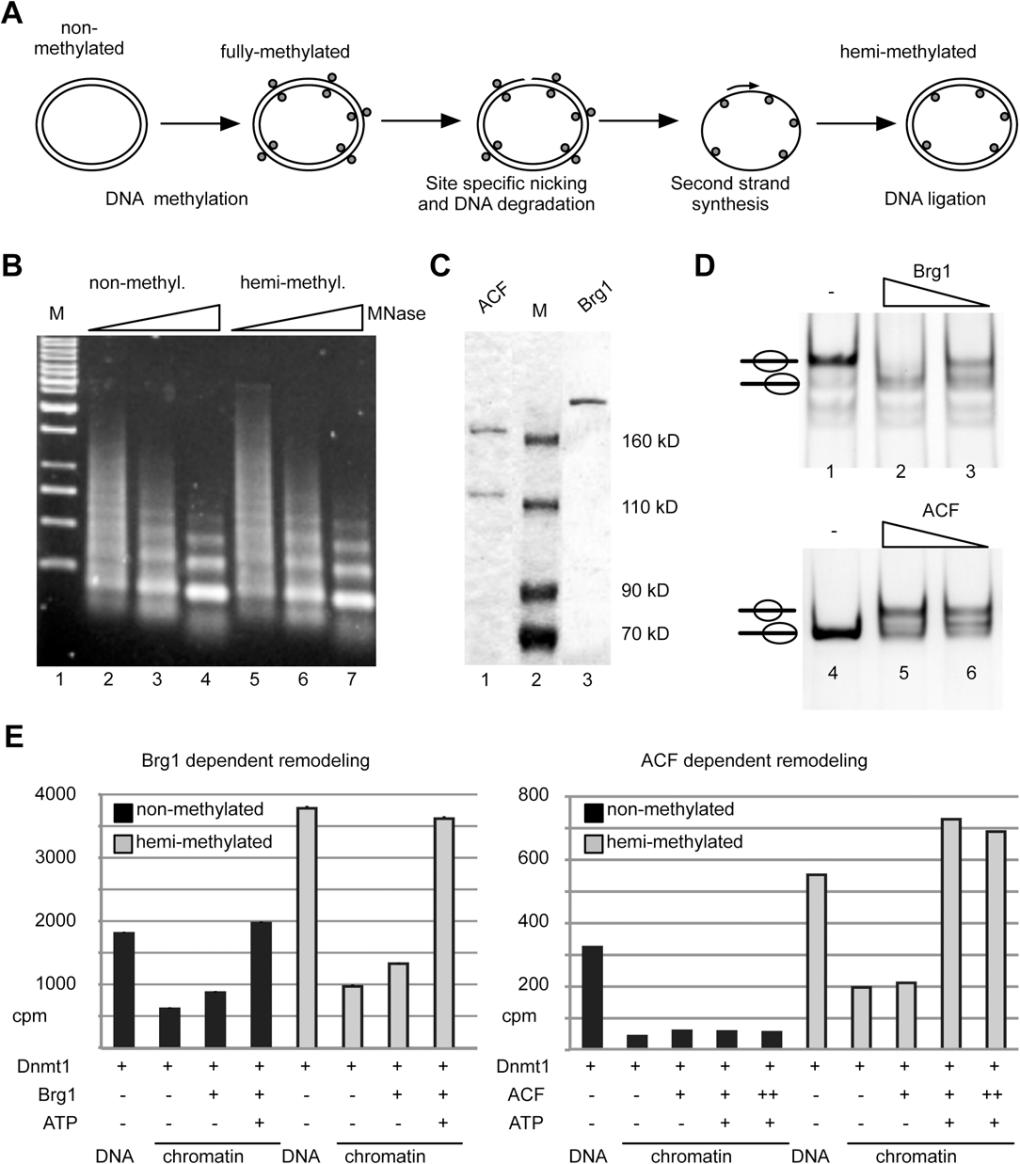
DNA methylation in the context of nucleosomal arrays and chromatin remodeling enzymes. **(A)** Schematic illustration of the method used to generate hemi-methylated DNA. **(B)** Non-methylated and hemi-methylated nucleosomal arrays generated by salt gradient dialysis were analyzed by partial MNase digestion for 10 to 90 sec (lanes 2–4 and 5–7). The purified DNA was analyzed by agarose gel electrophoresis and ethidium bromide staining. The DNA marker is loaded in lane 1 (M). **(C)** SDS-PAGE showing the purified, recombinant chromatin remodeling enzymes ACF and Brg1. **(D)** Testing the activity of the purified remodeling enzymes in nucleosome remodeling assays. Brg1 was incubated with a nucleosomal substrate positioned at the center of the DNA fragment (lanes 1 to 3). ACF was tested with a nucleosome positioned at the border of a DNA fragment of 208 bp in size (lanes 4–6). Reactions contained 1mM ATP and were stopped after 60 min by the addition of competitor DNA. Nucleosome positions were analyzed by native PAGE. **(E)** Analysis of nucleosome remodeling and Dnmt1 dependent DNA methylation on nucleosomal arrays. Non-methylated (black bars) or hemi-methylated (grey bars) nucleosomal arrays were incubated with Dnmt1, ATP, Brg1 and ACF as indicated. The incorporation of [^3^H]-labeled CH_3_ was determined by scintillation counting, shown as counts per minutes (cpm).

Next we asked whether nucleosomal arrays and especially hemi-methylated nucleosomal arrays represent Dnmt1 substrates. First, we generated a hemi-methylated, circular plasmid DNA as template for the chromatin assembly reactions. The plasmid was first fully methylated with SssI methyltransferase, then a single strand break was introduced with a nicking enzyme and the second, non-methylated DNA strand was produced by a primer extension reaction and subsequent ligation (Fig 6A). Nucleosomal arrays were reconstituted by the salt dialysis method, and the assembly efficiency and the quality of the arrays was verified by partial MNase digestion (Fig 6B). The MNase ladder and the kinetics of digestion shows that both, the non- and hemi-methylated DNA templates, were fully reconstituted into chromatin and of high quality. To study Dnmt1 dependent DNA methylation in the presence of chromatin remodeling enzymes we used the recombinant nucleosome remodeling machines Brg1 and ACF (Fig 6C). Both enzymes are capable to mobilize nucleosomes as shown in the nucleosome mobility assays (Fig 6D) [37]. According to their described remodeling features, Brg1 activity was examined on a mono-nucleosomal template with symmetric DNA linkers, whereas ACF was tested on a nucleosomal substrate with the nucleosome positioned at the border of the DNA fragment [38]. Both machines are highly active and moved the histone octamer to other positions on the DNA template (Fig 6D). Dnmt1 dependent DNA methylation was investigated on non-methylated and hemi-methylated nucleosomal arrays, in the presence or absence of Brg1 and ACF respectively (Fig 6E). DNA methylation was quantified after incorporation of the ^3^H-labeled methyl group by scintillation counting. Free DNA is efficiently methylated and, as expected, we observed increased methylation efficiency on the hemi-methylated DNA (Fig 6E, DNA). The assembly of the DNA into chromatin results in a strong inhibition of DNA methylation, only allowing residual DNA methylation. The nucleosomal arrays do apparently not present efficient substrates of Dnmt1, suggesting that the linker DNA is masked in the folded array. Differences in the levels of total DNA methylation counts are due to the biological repeats of the chromatin assembly protocols. However, the qualitative results of the individual experiments are very well comparable with each other (Fig 6E, S4 Fig). Repression of DNA methylation on hemi-methylated chromatin is overcome by the presence of active chromatin remodeling enzymes as they make the DNA accessible for Dnmt1 (Fig 6E, S4 Fig). These experiments show for the first time the interplay between Dnmt1 and remodeling enzymes *in vitro*, pinpointing to the important role of chromatin remodelers in regulating DNA methylation *in vivo*. Interestingly, we observed striking differences between the ATPases Brg1 and ACF. Whereas Brg1 increases the DNA methylation efficiency on both, the non- and hemi-methylated nucleosomal template, ACF is only able to stimulate DNA methylation on hemi-methylated chromatin (Fig 6E and S4 Fig). The effects of Brg1 potentially depend on the previously shown nucleosome disruption activity of the enzyme that allows the access to the nucleosomal CpG sites [39,40]. In contrast, it was shown that ACF serves as an chromatin assembly factor and stabilizes nucleosomes, explaining the in-accessibility of the non-methylated chromatin substrate [41,42]. However, strong ACF dependent increase in DNA methylation efficiency on hemi-methylated DNA suggests a functional interaction of Dnmt1 and ACF on hemi-methylated chromatin to open the nucleosomal array. Indeed, cooperative binding of Dnmt1 and Snf2h to nucleosomes as well as a functional role of ACF in the establishment and maintenance of heterochromatin are reported [43,44]. Our data now suggest a specific role for ACF in rendering hemi-methylated CpG sites accessible for Dnmt1.

## Discussion

The biological importance of DNA methylation to normal mammalian development has been demonstrated [45–47]. Only a few biochemical studies addressed DNA methylation within the context of chromatin, giving a limited understanding of the functional and mechanistic contributions of Dnmt1 towards maintaining and establishing genomic methylation. In this study we examined the binding characteristics of Dnmt1 to DNA and mono-nucleosomes exhibiting DNA linkers of varying length, the methylation of nucleosomes as well as nucleosomal arrays and the effect of Brg1 and ACF on DNA methylation. Our data reveal the importance of accessible linker DNA to enable nucleosomal binding and the inability of Dnmt1 to methylate DNA within the nucleosome core. Nucleosomal arrays present bad DNA methylation substrates, potentially by the wrapping and masking of linker DNA in higher order structures, thus requiring the activity of remodeling enzymes to enable DNA methylation in chromatin.

In order to assess the mechanism of DNA methylation in chromatin we first studied the binding of Dnmt1 to nucleosomal DNA. Competitive binding experiments were performed with nucleosomes having symmetric or asymmetric DNA linkers of varying length. We observed that Dnmt1 rather prefers to bind to nucleosomes with long asymmetric linkers (77 bp) and does not stably interact with nucleosomes having shorter asymmetric DNA linkers or the nucleosome core (Fig 1). However, Dnmt1 does interact with nucleosomes having short (22 to 40bp), but symmetric DNA linkers (Fig 1), suggesting a distinct binding mode. Interestingly these DNA linker lengths reflect the linker sizes present in cellular chromatin. The nucleosome repeat length (NRL) varies between tissues and within genomic regions, correlating with the overall activity of genomic regions. Highly active cells generally have shorter NRLs than resting cells, reaching from 178 bp in CHO cells to 207 bp in chicken erythrocytes [48]. CHO cells have a mean linker length of 31 bp that would allow efficient binding of Dnmt1 to nucleosomes.

The facilitated binding of Dnmt1 to nucleosomal DNA in the presence of symmetric linkers suggests a cooperative binding mode of Dnmt1, probably as a homotypic multimer in solution. However, in a different study Dnmt1 was shown not to bind cooperatively to DNA [49]. The experiments in this study were performed with DNA duplexes of 30 bp, which, according to our results, are too short to reveal cooperative binding. Hence, the authors could not observe cooperative binding. In contrast, our experiments show highly cooperative binding, and at conditions of DNA excess, the longest DNA molecules are preferentially bound (Fig 2). MST measurements with free DNA show a Hill coefficient of about 2.7, and the structural analysis of Dnmt1 clearly shows that Dnmt1 forms stable dimers in solution (S3 Fig). The gelfiltration and co-immunoprecipitation assays suggest that dimerization requires the TS domain of Dnmt1, confirming previous data by the Leonhardt group [50]. Our data indicate that on longer DNA fragments both Dnmt1 molecules of the dimer bind to DNA, implying that the binding of a single Dnmt1 molecule to a 30 bp long DNA fragment would be exceeded. The existent DNA length sensing activity also suggests that, once the Dnmt1 dimer is bound to DNA, it presents a preferential binding site for additional Dnmt1 molecules and/or the nucleoprotein-complex has reduced DNA OFF-rates. In addition, we show that the binding affinity of Dnmt1 does not change significantly when comparing non-methylated and hemi-methylated substrates (Fig 3). As a consequence it may be that the enzymatic properties of Dnmt1 are changed by the methylated residue and not by the binding event itself. The requirement of symmetric linkers presenting two DNA binding platforms fits perfectly to the observation of a dimeric form of Dnmt1. Each Dnmt1 molecule in the dimer could contact one linker and thereby embrace the nucleosome core. Indeed our footprinting experiments with isolated Dnmt1-nucleosome complexes revealed the simultaneous occupation of both DNA linkers (Fig 4C). Due to the geometry of the nucleosome, the result implies that Dnmt1 is located over the dyad axis of the nucleosome. Such a binding mode would even stabilize the nucleosome as it would reduce nucleosomal breathing and mobility of the DNA at the entry/exit site [51]. Accordingly, we see no disruption of the octameric complex upon Dnmt1 binding (Fig 1E).

To date two studies, showing contradicting results, addressed the DNA methylation of nucleosomal DNA by Dnmt1. Whereas Gowher and colleagues demonstrate that Dnmt1 is able to methylate nucleosomal sequences, Okuwaki and Verreault see a repression of DNA methylation [15,17]. In our DNA methylation assays we used purified nucleosomes, being positiond at a unique translational site and devoid of contaminating free DNA. As suggested by the ^3^H-SAM methyltransferase assay, the nucleosome core seems to be refractory to DNA methylation (Fig 5C). For a proper realization of the experiments, we used nucleosomal substrates with linker DNA that can be efficiently bound by Dnmt1, having CpG dinucleotides at regular spacing and having the accessible linker DNA as a reference. High resolution analysis of DNA methylation by bisulfite sequencing revealed that, like in the radioactive assay, the nucleosomal DNA is almost free of DNA methylation, whereas the linker DNA is efficiently methylated. The experiments clearly show the inaccessibility of the nucleosomal DNA for Dnmt1 (Fig 5D). Our study reveals a much stronger protection as previously documented, which may be due to the highly pure nucleosome fraction that has been used. Still, close to the entry/exit sites reduced but significant levels of DNA methylation were detectable. This suggests, as also stated by Okuwaki and Verreault, that CpGs in specific orientations to the histone surface are partially accessible [17]. Our results are supported by recent findings *in vivo*, showing that mainly the linker DNA is methylated, whereas the nucleosomal core is depleted of DNA methylation [18,19]. This also implies that the chromatin structure *in vivo* is more static than previously anticipated, suggesting the existence of active mechanisms to render the regulatory nucleosomes accessible for DNA methylation.

Chromatin remodeling complexes consist of a central ATPase unit and 1 to 20 additional subunits [52]. These complexes are highly abundant in the cell and use the energy of ATP hydrolysis to mobilize, stabilize, space or evict nucleosomes. Thereby these machines organize chromatin structures and regulate the access to nucleosomal DNA. Hundreds of different remodeling complexes with distinct functions exist in the cell that specifically recognize and remodel nucleosomes [8,38]. Brg1 belongs to the class of remodeling enzymes that leads to the opening of chromatin structures, whereas ACF is rather a nucleosome stabilizing entity that is predominantly associated with the formation of compact chromatin [36,52]. We used these opposing remodeling activities to study Dnmt1 dependent DNA methylation on nucleosomal arrays. We created a circular, hemi-methylated DNA substrate that was efficiently assembled into nucleosomal arrays and performed DNA methylation assays in the presence or absence of the remodeling enzymes (Fig 6). In the case of Brg1, as expected, the data clearly shows the requirement of ATP dependent nucleosome remodeling to modify both, non-methylated and hemi-methylated, types of chromatin. In contrast, ACF shows a functional interaction with the DNA methyltransferase in that DNA methylation on non-methylated chromatin is repressed, whereas Dnmt1 is active on the hemi-methylated nucleosomal substrate (Fig 6). Our findings suggest that chromatin remodeling complexes in cooperation with the DNA methyltransferases regulate the methylation of the underlying DNA. Our data would correlate with the specific function of ACF/CHRAC in DNA replication and repair, where full DNA methylation signals have to be established [53–56]. This cooperation suggests that in cells remodeling enzymes regulate the accessibility of DNA for DNA methylation, thereby determining the loci to be modified.

## Methods

### Plasmids

The plasmids pPCRScript_slo1-gla75, pGA4 BN601-m1, pMA BN601mod rDH70_Cless were used for PCR amplification of DNA fragments for chromatin reconstitution (S1 Table) [18].

### Protein purification

Baculovirus carrying N-His Dnmt1 was prepared as described [57]. 2.0x10^8^ Sf21 insect cells were infected for 48–60 h. Sf21 insect cells were resuspended in 10 ml lysis buffer (20 mM Tris, pH 7.6, 250 mM KCl, 1 mM imidazole, 1.5 mM MgCl_2_, 0.5 mM EGTA, 10% glycerol, 1% NP40, 2 mM ß-mercaptoethanol and lysed by three repeated freeze-thaw cycles and subsequently treated with five strokes each of A-type and B-type pestle in a dounce homogenizer. Cells were treated by sonification with a Branson Sonifier 250D (3x for 30 s 50% amplitude, 50% duty cycle) following clearance by centrifugation (30 min, 20,000 g, 4°C). The supernatant was subsequently used for protein purification with Ni-NTA beads (Qiagen) according to manufacturers instruction. Brg1 and ACF proteins were purified as described [38].

### Microscale thermophoresis

Binding affinities of Dnmt1 to hemi- and unmethylated double stranded (ds) DNA were measured by microscale thermophoresis (MST) [34,35]. In brief, complementary AIR60 oligonucleotides (S2 Table; each 500 pmol) were hybridized in binding buffer (200 mM Tris/Cl pH 7.4; 20 mM MgCl_2_; 500 mM NaCl) to generate ds hemi-methylated or non-methylated DNA, labeled with the fluorescent cyanine dyes Cy5 or Cy3. The hybridization reactions were analyzed by native polyacrylamide gel electrophoresis (PAGE). Dnmt1 was concentrated by ultrafiltration using a 10K centrifugal filter device (Amicon). Concentrated Dnmt1 was analyzed by sodium dodecyl sulfate polyacrylamide gelelectrophoresis (SDS-PAGE). A mixture of 25 nM of each the hemi- and the non-methylated ds DNA, one Cy5 and the other Cy3 labeled, were incubated with increasing concentrations of Dnmt1 (300 nM—9.4 μM) in reaction buffer (10 mM Tris/Cl, 150 mM KCl, 0.75 mM MgCl_2_, 0.25 mM EGTA, 5% glycerol, 0.05% NP40) at 25°C for 15 min, in the presence or absence of S-adenosyl methionine (SAM, 160 μM; NEB). Measurements were done on a Monolith NT.115 device (Nanotemper Technologies). Plotting and curve fitting were done with Sigmaplot 12.5 (Systat Software). The Hill equation was applied for curve fitting.

### Nucleosome reconstitution by salt gradient dialysis

Chromatin assembly of DNA fragments (S1 Table) with purified histone octamers from chicken blood using the salt dialysis technique was performed as described previously[58]. A typical assembly reaction (50 μl) contained 5.0 μg DNA, varying amounts of histone octamer, 200 ng/μl bovine serum albumin (BSA), and 250 ng competitor DNA in high salt buffer (10 mM Tris pH 7.6, 2.0 M NaCl, 1.0 mM EDTA, 0.05% NP-40, 2.0 mM ß-mercaptoethanol). The salt was continuously reduced to 200 mM NaCl during 16–20 hours and finally dialyzed to 50 mM NaCl. The quality of the assembly reaction was analyzed on a 5.0% native polyacrylamide gel followed by ethidium bromide staining.

### Electromobility shift assay and nucleosome mobility assay

Electromobility shift assays with Dnmt1 were performed in 20 μl reaction volume in 20 mM Tris pH 7.6, 30 mM KCl, 5.0 mM EDTA, 1.0 mM DTT, 5.0 μM SAM and 20% glycerol [18]. Reconstituted nucleosomes (50 nM each) were incubated with increasing concentrations of Dnmt1 (100 nM– 0.5 μM) for 15 min at 26°C to allow complex formation. Reactions were put on ice and analyzed on a 5.0% native polyacrylamide gel.

The stability of the Dnmt1-mononucleosome complex was analyzed by competition experiments. 100–500 ng competitor DNA (pCMV14 plasmid DNA) were added to those reactions in which the formation of the mononucleosome was saturated with Dnmt1 (as monitored by native PAGE), and stability was analyzed on a native polyacrylamide gel.

Analysis of Dnmt1 binding to small DNA fragments was carried out as followed: Dnmt1 (0.1–0.5 μM) were titrated to the DNA substrate (65–125 ng ultra low range DNA ladder (Fermentas). The reaction was incubated for 30 min at 26°C in binding buffer and DNA-protein complexes were analyzed on a 15% native polyacrylamide gel.

Nucleosome remodeling reactions were performed as described [38]. Briefly, reaction mixes in RB90 buffer (20 mM Tris pH 7.6, 1.5 mM MgCl_2_, 1.0 mM EDTA, 10% glycerol, 90 mM KCl, 1.0 mM DTT) supplemented with 1 mM ATP and 200 ng/μl BSA containing 200 ng (1.0 picomol) reconstituted nucleosomes and 50–200 ng (0.5–2.0 picomol) Brg1 or ACF were incubated for 90 min at 26°C. Remodeling reactions were stopped by the addition of competitor DNA and nucleosome positions were analyzed on native polyacrylamide gels.

### DNaseI footprinting

DNaseI footprinting was performed with a purified, fluorescently labeled 301 bp nucleosomal template (77-NPS-77), harboring the 601 nucleosome positioning sequence. The footprint was analyzed by capillary electrophoresis. 25 nM naked or nucleosomal 77-NPS-77 template was incubated with increasing concentrations of Dnmt1 (0 to 250 nM) in binding buffer (3 mM Tris/Cl, 50 mM KCl, 0.25 mM MgCl_2_, 80 μM EGTA, 5 mM CaCl_2_) for 30 min allowing formation of the nucleosome-protein complex. The samples were incuabted with DNaseI and the reaction was stopped by the addition of EDTA and directly separated on a 5% native polyacrylamide gel. The bands representing the DNA, nucleosomes and Dnmt1-nucleosome complexes were excised and the DNA was purified. Approximately 250 pM purified DNA were loaded onto a capillary electrophoresis instrument. The injection was 2 kV and injection time 15 s. The intensity was measured in relative fluorescence units (RFU). As size standard a 1:2 mixture of GeneScan- LIZ120 and LIZ500 was used.

### *In vitro* methyltransferase assay

A typical methyltransferase reaction (50 μl) contained 100 nM Dnmt1, free or nucleosomal DNA template at 500 nM and 5000 nM CpG sites respectively, 200 ng/μl BSA and 160–320 nM ^3^H-SAM (GE Healthcare, TRK581-250UCi, 9.25 MBeq with 1.0 mCi/ml 63.0 Ci/mmol) in methyltransferase buffer (20 mM Tris, pH 7.6, 1.0 mM EDTA, 1.0 mM DTT). The reaction was started with the addition of DNA, incubated at 37°C for 10–60 min, and stopped by the addition of 10 μl of 10 mM SAM (Sigma). The reaction was spotted on DE81 filter (Whatman), washed three times with 0.2 M NH_3_HCO_3_, once with water and ethanol following drying and scintillation counting.

### Bisulfite conversion and analysis of CpG site methylation

Site-specific DNA methylation analysis was performed with the C91-NPS2-C104 DNA fragment (342 bp, 27 CpG sites, non-methylated) as free DNA or fully reconstituted mononucleosome. DNA methylation reactions containing 120 nM Dnmt1 were carried out in DNA methyltransferase buffer (20 mM Tris, pH 7.6, 1.0 mM EDTA, 1.0 mM DTT), 200 ng/μl BSA, 250 nM SAM (Sigma) and free DNA or mononucleosomes (16 nM CpG sites). The reactions were incubated for 4 hours at 37°C following heat inactivation at 65°C for 20 min. DNA methylation reactions were processed according to the Epitect bisulfite conversion kit (Qiagen). The upper strand (+) and the lower strand (-) of the bisulfite converted DNA were PCR-amplified with primer pairs MF81/82 and MF112/113 (S2 Table).

PCR fragments were cloned into the pGEM-T-EASY vector (Promega) according to the manufacturer’s instructions, and the DNA from positive clones was sent for sequencing. Analysis and quality control of bisulfite converted DNA was done with the BiQ ANALYZER software (http://biq-analyzer.bioinf.mpi-inf.mpg.de/) provided by C. Bock [59]. The DNA methylation frequency of each CpG site was plotted against the respective CpG dinucleotides of the DNA sequence.

### DNA fragments

DNA fragments containing a modified 601 nucleosome positioning sequence [32] (referred to as NPS, 142 bp, 3 CpG sites) are flanked by a partial murine rDNA promoter sequence (80 bp) on the left and a partial *Drosophila* HSP70 promoter sequence (85 bp) on the right. By using different combinations of oligonucleotides, symmetrical and asymmetrical linker DNA of variable length, relative to the NPS sequence, can be generated. In addition, two other DNA fragments were designed for DNA methylation studies (NPS2 and C91-NPS2-C104). Both carry a modified 601 nucleosome positioning sequence but differ in their CpG content of their flanking DNA overhangs (S1 Table).

The DNA fragments were amplified by PCR and purified DNA fragments were subsequently used for nucleosome assembly reactions. Due to oligonucleotide annealing the NPS used consists of 142 bp instead of 147 bp.

### DNA sequences

NPS2 (150 bp, 15 CpG sites)

GATCCCGAATCCCGGTGCCGAGGCCGCTCAATTGGTCGTAGCAACGTCTAGCACCGCTTAAACGCACGTACGCGCTGTCCCCCGCGTTTTAACCGCCAAGGGGATTACTCCCTAGTCTCCAGGCACGTGTCAGATATATACAGCTAG

C91-NPS2-C104 (342 bp, 27 CpG sites)

GAATTGGGTACCAGATCTTTTGAGGTCCGGTTCTTTTCGTTATGGGGTCATATGTTTCGGCCACCTCCCCATGGTACGACTTCCAGGTACGGATCCCGAATCCCGGTGCCGAGGCCGCTCAATTGGTCGTAGCAACGTCTAGCACCGCTTAAACGCACGTACGCGCTGTCCCCCGCGTTTTAACCGCCAAGGGGATTACTCCCTAGTCTCCAGGCACGTGTCAGATATATACAGCTAGCGACAAAGAAAACTCGAGAAATTTCTCGTAAGGCCGTTATTCTCTAGATTCGTTTTGTGACGCTCCCTCTCCGTACTAAGATCTGAGCTCCAGCTTTTGTTCCC

NPS (142 bp, 3 CpG sites)

GATCCAGAATCCTGGTGCTGAGGCTGCTCAATTGGTTGTAGCAAGCTCTAGCACTGCTTAAATGCATGTACGCGCGGTCCCCTGTGTTTTAACTGCCAAGGGGATTACTCCCTAGTCTCCAGGCATGTGTCAGATATATACA

77-NPS-77 (301 bp, 9 CpG sites)

ATCTTTTGAGGTCCGGTTCTTTTCGTTATGGGGTCATATGTTTGGGCCACCTCCCCATGGTATGACTTCCAGGTATGGATCCAGAATCCTGGTGCTGAGGCTGCTCAATTGGTTGTAGCAAGCTCTAGCACTGCTTAAATGCATGTACGCGCGGTCCCCTGTGTTTTAACTGCCAAGGGGATTACTCCCTAGTCTCCAGGCATGTGTCAGATATATACAGCTAGCTAGCAAAGAAAACTCGAGAAATTTCTCTTAAGGCCGTTATTCTCTAGATTCGTTTTGTGACTCTCCCTCTCTGTAC

## Supporting Information

S1 Fig Dnmt1 preferentially methylates hemi-methylated DNA. (PDF)

S2 Fig Illustration of the DNA substrate design used for the nucleosome binding assays. (PDF)

S3 Fig Dnmt1 forms dimers in solution via interaction with the TS domain of Dnmt1. (PDF)

S4 Fig Analysis of Dnmt1 methyltransferase activity on nucleosomal arrays in the context of chromatin remodeling. (PDF)

S1 Methods (DOCX)

S1 Table List of primers and templates used to generate the DNA fragments for nucleosome assembly. (PDF)

S2 Table List of oligonucleotides used in this study. (PDF)
