# Supplementary material for: Characterization of Dnmt1 Binding and DNA Methylation on Nucleosomes and Nucleosomal Arrays
Source: PLoS One. 2015 Oct 23;10(10):e0140076. doi: 10.1371/journal.pone.0140076 (PMC4619679; doi:10.1371/journal.pone.0140076)
Supplement: S1 Fig — (PDF) [file pone.0140076.s001.pdf]

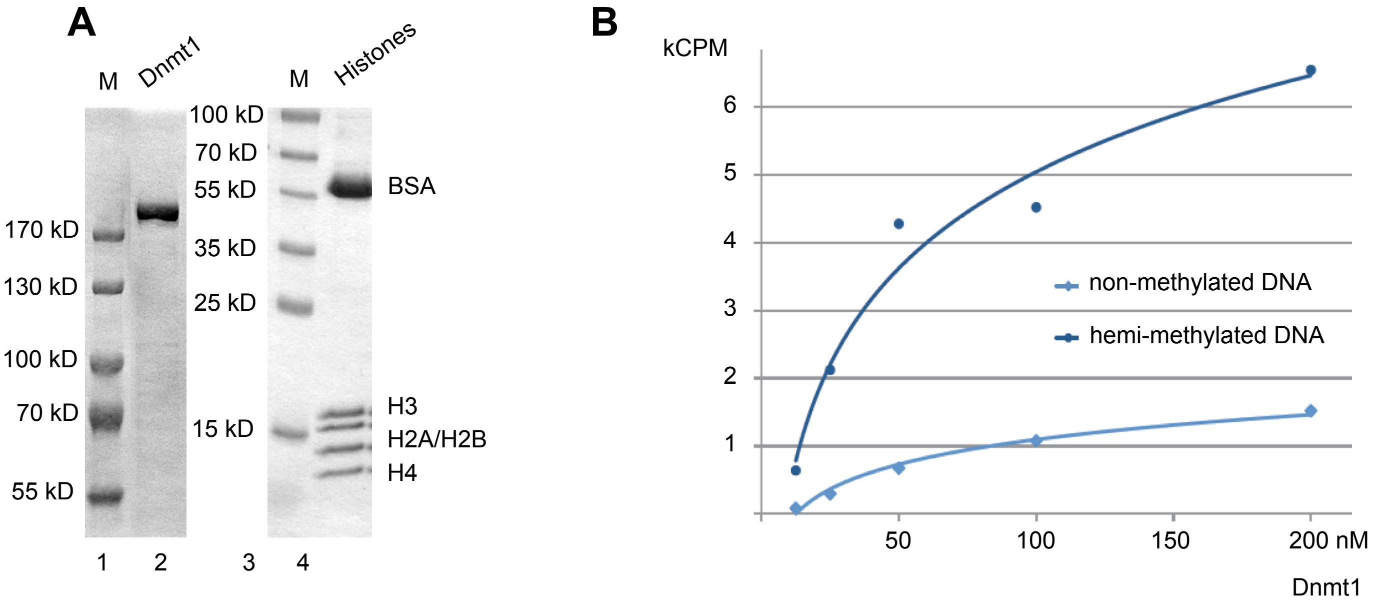

S1 Fig. Dnmt1 preferentially methylates hemi-methylated DNA. (A) Sodium dodecyl sulfate polyacrylamide gelelectrophoresis (SDS-PAGE) showing the purified, recombinant Dnmt1 (lane 2) and histone proteins (lane 4). The proteins were purified as described and analyzed by SDS-PAGE. **(B)** *In vitro* DNA methyltransferase activity assay using 12.5 – 200 nM Dnmt1, fixed DNA concentrations of 555 nM DNA and 411 nM [3H]-SAM, representing 5 pMol of CpG sites. The specific incorporation of [3H]-methyl groups is shown as kilo counts per minute (kcpm).
