# Supplementary material for: Characterization of Dnmt1 Binding and DNA Methylation on Nucleosomes and Nucleosomal Arrays
Source: PLoS One. 2015 Oct 23;10(10):e0140076. doi: 10.1371/journal.pone.0140076 (PMC4619679; doi:10.1371/journal.pone.0140076)
Supplement: S2 Fig — (PDF) [file pone.0140076.s002.pdf]

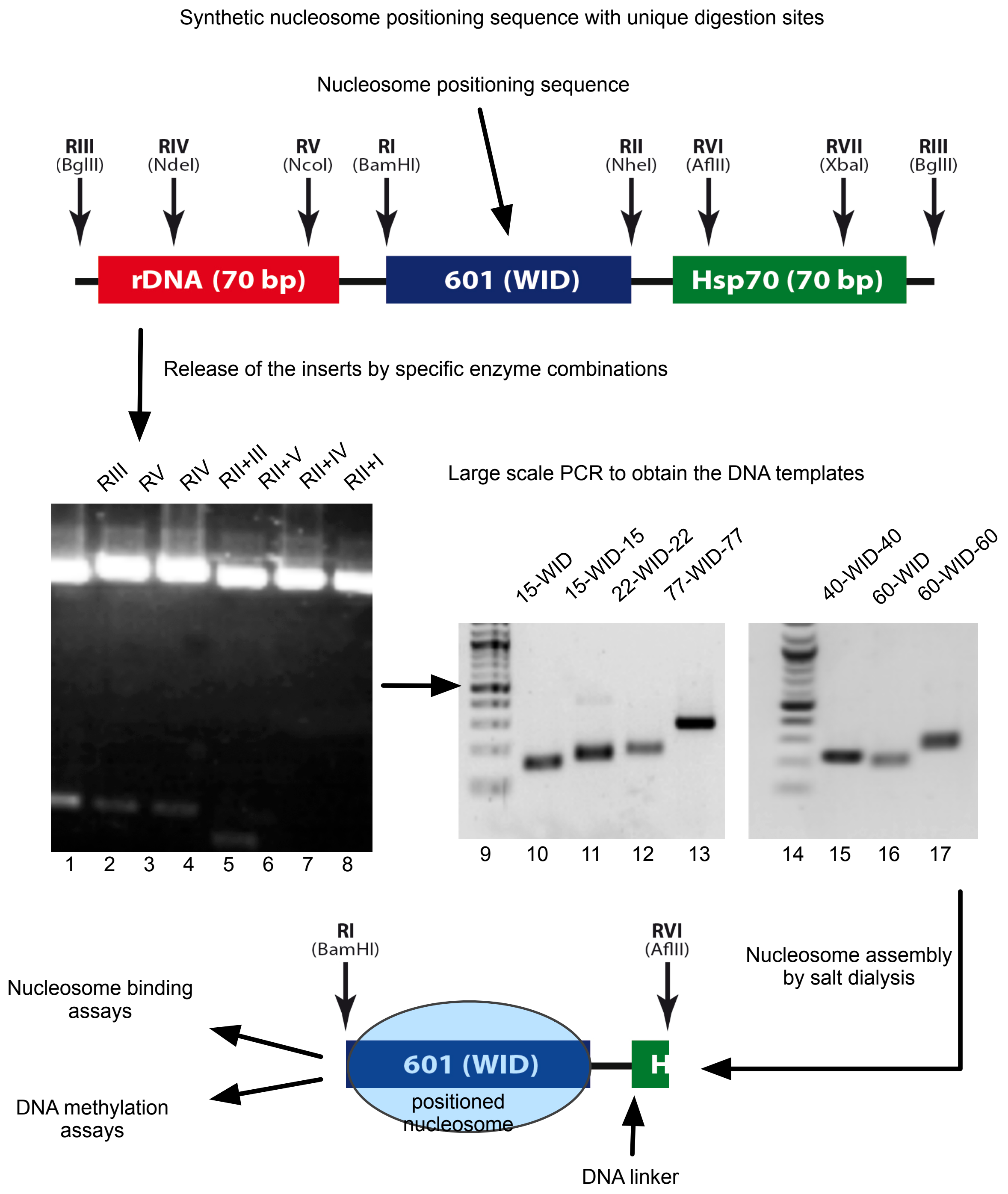

S2 Fig. Illustration of the DNA substrate design used for the nucleosome binding assays. The different DNA substrates were generated by the use of the respective restriction enzyme combinations and the plasmid pPCRScript_slo1-gla75. A scheme of the DNA templates and restriction enzyme cleavage sites is given. The 601 nucleosome positioning sequence is flanked by structured DNA elements originating from the murine rDNA (80 bp) and the *Drosophila* Hsp70 (85 bp) genes. By the combination of different restriction endonucleases, symmetrical and asymmetrical DNA overhangs of variable lengths were prepared and analyzed on a 1.3 % agarose gel. The different restriction endonucleases (RI-RV) used here are indicated on top (lanes 2-8). The resulting DNA fragments were amplified by PCR to generate the respective DNA substrates (lanes 9-17) for nucleosome assembly by salt gradient dialysis.
