# Supplementary material for: Characterization of Dnmt1 Binding and DNA Methylation on Nucleosomes and Nucleosomal Arrays
Source: PLoS One. 2015 Oct 23;10(10):e0140076. doi: 10.1371/journal.pone.0140076 (PMC4619679; doi:10.1371/journal.pone.0140076)
Supplement: S3 Fig — (PDF) [file pone.0140076.s003.pdf]

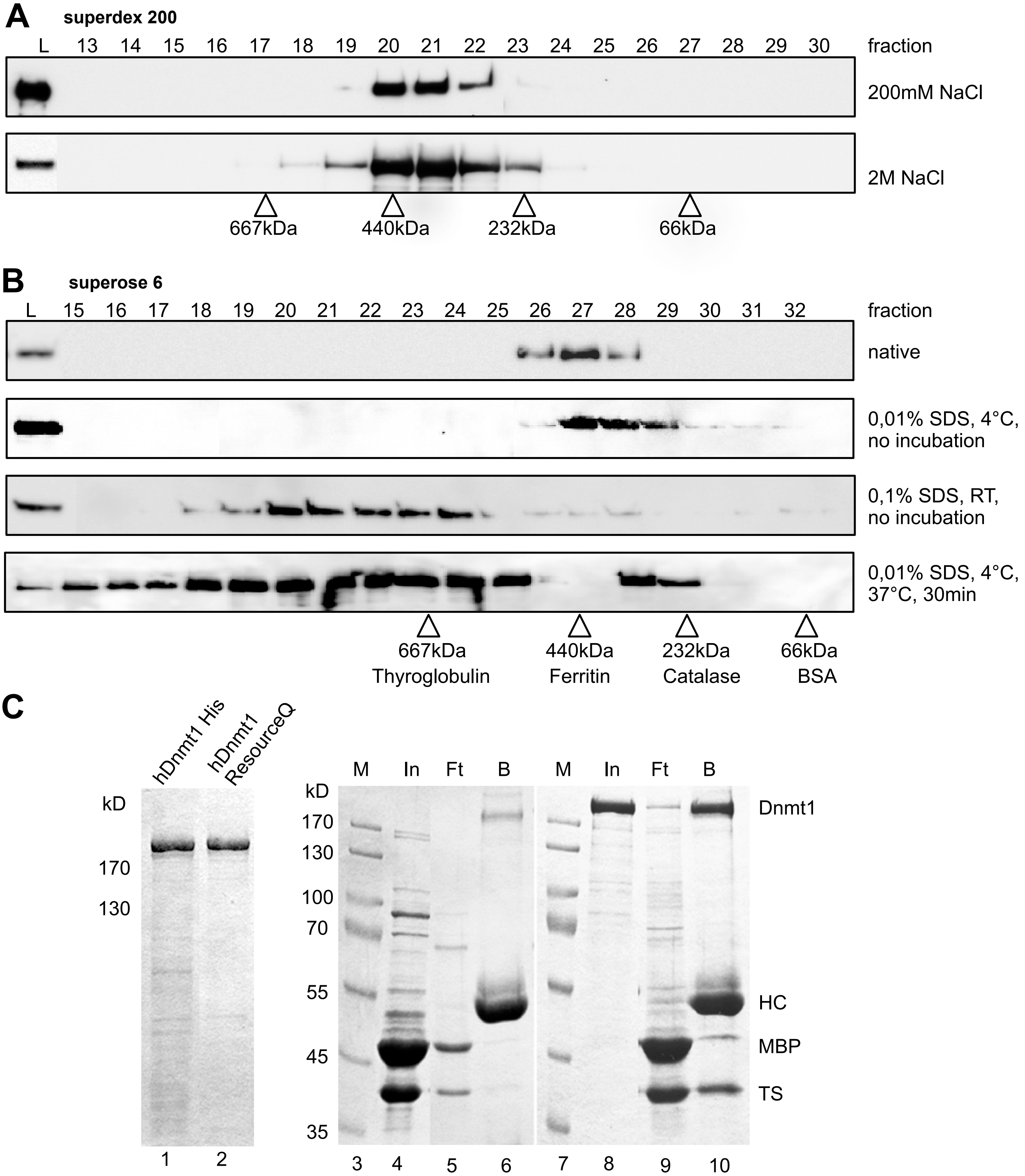

S3 Fig. Dnmt1 forms dimers in solution via interaction with the TS domain of Dnmt1.

A) Recombinant Dnmt1 was loaded on a superdex200 gelfiltration column (GE Healthcare) in running buffer (200mM NaCl/2M NaCl, 20mM Tris, pH 8.0; 1mM MgCl2, 10% glycerol). Upper panel. Dnmt1 in running buffer supplemented with 200mM NaCl. Lower panel. Dnmt1 in running buffer with 2M NaCl. **B)** Dnmt1 (30μg) was adjusted to the indicated SDS concentration and either incubated for 30min at 37°C or directly loaded on a superose6 gelfiltration column (GE Healthcare) in running buffer (150mM NaCl, 20mM Tris, pH 7.5; 1mM EDTA, 10% glycerol) supplemented with the indicated concentrations of SDS. For both columns, 500μl fractions were collected, TCA precipitated and subjected to SDS-PAGE and Western Blot analysis (detection with the Dnmt1 specific antibody 2G3). Molecular weight standards and fractions are indicated. **C)** Dnmt1 co-immunoprecipitates the TS domain. Recombinant Dnmt1 was expressed in insect cells, purified first by Ni-NTA affinity chromatography and second by chromatography on ResourceQ material (GE Healthcare) (lanes 1 and 2). Purified MBP-TS was cleaved with TEV Protease (lane 4) and incubated with the Dnmt1 specific antibody 2E8 coupled to proteinG beads. 2% of the flowthrough and 50% of the beads were loaded on a 6% SDS-PAGE (lanes 5 and 6). Recombinant Dnmt1 (lane 8) was incubated with a 50 fold excess of the TS domain for 1h at 30°C. The reaction was incubated with the 2E8 antibody coupled to proteinG beads and 10% of the flowthrough and 50% of the beads were loaded on a 6% SDS-PAGE (lane 9 and 10). Proteins were stained with Coomassie Blue. The positions of Dnmt1, the antibody heavy chain (HC), the Maltose Binding Protein (MBP) and the human TS domain are indicated.
