# Supplementary material for: Characterization of Dnmt1 Binding and DNA Methylation on Nucleosomes and Nucleosomal Arrays
Source: PLoS One. 2015 Oct 23;10(10):e0140076. doi: 10.1371/journal.pone.0140076 (PMC4619679; doi:10.1371/journal.pone.0140076)
Supplement: S4 Fig — (PDF) [file pone.0140076.s004.pdf]

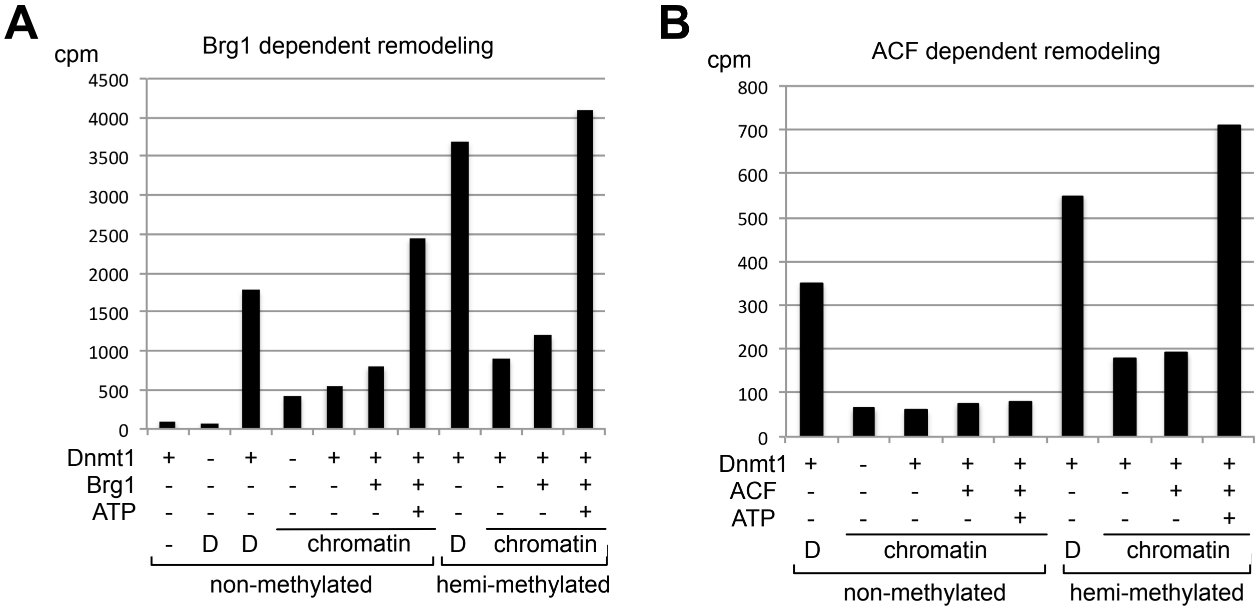

S4 Fig. Analysis of Dnmt1 methyltransferase activity on nucleosomal arrays in the context of chromatin remodeling. Non-methylated or hemi-methylated free DNA or nucleosomal arrays were incubated with Dnmt1, ATP, Brg1 and ACF as indicated. The incorporation of [^{3}H]-labeled CH_3 was determined by scintillation counting. (D = free DNA). (A) Brg1 increases the DNA methylation efficiency on the non - and hemi-methylated nucleosomal substrate. (B) ACF stimulates DNA methylation only on hemi-methylated nucleosomal arrays.
