# Supplementary material for: Characterization of Dnmt1 Binding and DNA Methylation on Nucleosomes and Nucleosomal Arrays
Source: PLoS One. 2015 Oct 23;10(10):e0140076. doi: 10.1371/journal.pone.0140076 (PMC4619679; doi:10.1371/journal.pone.0140076)
Supplement: S1 Methods — (DOCX) [file pone.0140076.s005.docx]

## S1 Methods:

**Gelfiltration chromatography.** For gelfiltration runs, superose6 or superdex200 gelfiltration columns (GE Healthcare) were washed with two column volumes water and equilibrated with running buffer. Separation was performed in gelfiltration buffer (20mMTris pH7.5, 150mM NaCl, 1mM EDTA, 1mM DTT) with a flow rate of 0.5ml/min. Gelfiltration columns were calibrated with protein standards that meet the requirement of being spherical and inert. The Dnmt1 specific antibody 2G3 (rat, monoclonal) was kindly provided by E. Kremmer (Helmholtz Gesellschaft).

**Co-immunoprecipitation.** 100μl of proteinG bead slurry were washed three times with 850μl IP buffer (50mM Tris pH 7.5, 150mM NaCl, 0.1mM EDTA, 0.05% NP-40). Equilibrated proteinG beads were incubated with the protein mixture at 4°C for 2 hours. Beads were washed three times and resuspended in 50μl HU buffer after removal of the supernatant. The suspension was heated to 65°C for 10 min and subjected to SDS PAGE analysis. The Dnmt1 specific antibody 2E8 (rat, monoclonal) was a gift from E. Kremmer (Helmholtz Gesellschaft)
