# Supplementary material for: Characterization of Dnmt1 Binding and DNA Methylation on Nucleosomes and Nucleosomal Arrays
Source: PLoS One. 2015 Oct 23;10(10):e0140076. doi: 10.1371/journal.pone.0140076 (PMC4619679; doi:10.1371/journal.pone.0140076)
Supplement: S1 Table — (PDF) [file pone.0140076.s006.pdf]

S1 Table. List of primers and templates used to generate the DNA fragments for nucleosome assembly

DNA fragments	Primer (forward)	Primer (reverse)	DNA template	length
NPS	AP7	AP8	pPCRScript_slo1-gla75	142
22-NPS	AP3	AP8	pPCRScript_slo1-gla75	164
22-NPS-22	AP3	AP13	pPCRScript_slo1-gla75	191
40-NPS	AP5	AP8	pPCRScript_slo1-gla75	182
40-NPS-40	AP5	AP14	pPCRScript_slo1-gla75	227
77-NPS	AP1	AP8	pPCRScript_slo1-gla75	219
77-NPS-77	AP1	AP15	pPCRScript_slo1-gla75	301
NPS2	MF124	MF125	pGA4 BN601-m1	150
C91-NPS2-C104	MF79	MF80	pGA4 BN601-m1	342
