# Supplementary material for: Characterization of Dnmt1 Binding and DNA Methylation on Nucleosomes and Nucleosomal Arrays
Source: PLoS One. 2015 Oct 23;10(10):e0140076. doi: 10.1371/journal.pone.0140076 (PMC4619679; doi:10.1371/journal.pone.0140076)
Supplement: S2 Table — (PDF) [file pone.0140076.s007.pdf]

S2 Table. List of oligonucleotides used in this study

Name	Sequence
AP1	ATCTTTTGAGGTCCGGTTCTTT
AP3	CATGGTATGACTTCCAGGTATGG
AP5	ATGTTTGGGCCACCTCCCC
AP7	GATCCAGAATCCTGGTGCTGAG
AP8	TGTATATATCTGACACATGCCTGGA
AP13	TTTCTCGAGTTTTCTTTGCTAGCT
AP14	TAACGGCCTTAAGAGAAATTTCT
AP15	GTACAGAGAGGGAGAGTCACAAAAC
MF79	GAATTGGGTACCAGATCTTTTGAG
MF80	GGGAACAAAAGCTGGAGCTC
MF81	GAATTGGGTATTAGATTTTTTGAGGTT
MF82	AAAAACAAAAACTAAAACTCAAATCTTAATA
MF112	GGGAATAAAAGTTGGAGTTTAGATTTTA
MF113	AAATTAAATACCAAATCTTTTAAAATCC
MF124	GATCCCGAATCCCGGTG
MF125	CTAGCTGTATATATCTGACACGTGCC
AIR up 60 Cy5	/5CY5/TGCGGAATCGTCTAACGCGTGGAATCGTCCGATTGAGGTCGCGATC TCGAATCACGGTGC
AIR down 60	GCACCGTGATTCGAGATCGCGACCTCAATCGGACGATTCCACGCGTTAGACG ATTCCGCA
AIR down 60 me	GCACZGTGATTZGAGATZGZGACCTCAATZGGAZGATTCCAZGZGTTAGAZG ATTCZGCA
	Z = methylated cytosine
